# Mentoring Top Leadership Promotes Organizational Innovativeness through Psychological Safety and Is Moderated by Cognitive Adaptability

**DOI:** 10.3389/fpsyg.2017.00318

**Published:** 2017-03-02

**Authors:** James H. Moore, Zhongming Wang

**Affiliations:** School of Management, Zhejiang UniversityHangzhou, China

**Keywords:** mentoring, innovativeness, cognitive adaptability, psychological safety, entrepreneurship, change, culture

## Abstract

Mentoring continues to build momentum among startups and established enterprises due to its positive impact on individuals and organizations. Unlike previous studies, this research focuses on mentoring higher level leadership, such as the CEO, and demonstrates its unique relationship to organizational innovativeness. Our sample included 200 mentored executives and entrepreneurs who personally identify and exploit opportunities. Our findings confirm that mentoring top leaders positively relates to their perceived innovativeness of the organization and that the relationship is mediated by these leaders’ perception of psychological safety within the organization. Our findings also confirm that the relationship is negatively moderated by these leaders’ cognitive adaptability. The reliability and validity of the results have been proved by using confirmatory factor analysis and advanced regression analytics. As a result, this work demonstrates the value of mentoring top leadership and advocates the importance of establishing a psychologically safe environment to inspire not only top leadership to try new avenues but also for all those within the organization to speak up and speak out. Additionally, our findings encourage organizations to proactively and selectively prioritize mentoring among top leadership, taking into account their differing levels of cognitive adaptability. Finally, further research could focus on how to provide greater support for mentors of higher level leaders.

## Introduction

Whether organizations are new or well established, they must be innovative and able to change in order to survive and thrive (Goyal and Pitt, 2007; Chesbrough, 2013). However, organizations do not just operate by themselves—they are powered and led by people. Leadership is one of an organization’s greatest assets and understandably needs to be developed. Since the ancient Greek times and even in our day mentoring has been identified as an innovation in management (Odiorne, 1985) and is considered an effective way to transfer entrepreneurial knowledge, skills, and attributes (Agbim et al., 2013; Wilbanks, 2013; St-Jean and Mathieu, 2015); however, this begs the question: Can mentoring promote something as tacit as innovativeness? This study is designed in part to shed light on this question as we explore the relationship between mentoring top leaders, such as a founder or CEO, and their perception of organizational innovativeness—the capacity and willingness to introduce new processes, products, or ideas within the organization (Damanpour, 1991; Hurley and Hult, 1998).

The relationship between mentoring top leaders and their perception of organizational innovativeness warrants further research for at least three reasons. First, mentoring and innovating share common ground. Both are a form of social interaction and contribute to the process of learning something new (Anthony, 2012; Capriati, 2013). However, neither the relationship nor the conditions under which it can be moderated or mediated have been confirmed or studied in depth. Previous studies have found that mentoring is related to positive results such as successful work outcomes for mid to lower level employees (Wanberg et al., 2006; Mitchell et al., 2015). However, none to date have focused on the link between the quality of mentoring higher level leaders and their perception of organizational innovativeness. Second, top leaders, such as CEO equivalents or entrepreneurs, are different from those at lower levels because of their high position, responsibility, ability to initiate change, and therefore potential influence on the organization (Papadakis and Barwise, 2002; Conger and Fulmer, 2003). Thus, understanding mentoring’s effects on developing this influential demographic is of great importance (de Janasz and Peiperl, 2015). Third, the mentoring of higher level leaders and lower level employees may or may not differ mechanically, but this study suggests that the value and overall impact of mentoring at higher levels does differ and therefore needs further study (Allen and Wanna, 2016).

Another important area of study is the intermediary process of the mentor influencing the mentee because this process facilitates mentoring outcomes (Baranik et al., 2010; Pan et al., 2011; Eby et al., 2013). For example, Chen et al. (2014) did a study on formal mentoring coming from a psychological perspective by exploring the mediating role of psychological safety on work attitudes. Our study furthers research on mediation in mentoring with psychological safety and links it to organizational innovativeness for two reasons. First, mentoring helps develop psychological safety within organizations (Kram, 1983; Kram and Isabella, 1985; McCauley and Young, 1993; Wang et al., 2010; Chen et al., 2014) and a climate of psychological safety can facilitate innovativeness within organizations (Edmondson, 2008); thus, psychological safety is sequentially an appropriate mediator because it can account for some of the influence mentoring has on innovativeness. Second, this is a new area of research because although there is support in the literature for strong connections between these variables individually, no empirical studies specifically link all three together (i.e., the quality of mentoring top leaders and their perception of organizational innovativeness via psychological safety). This research explores a significant way to potentially develop psychological safety from the top down and thereby enhance organizational innovativeness.

Finally, in spite of the accumulated research on the various effects of moderation on mentoring (Fleig-Palmer and Schoorman, 2011; Liang and Gong, 2013; Jyoti and Sharma, 2015; Son and Kuchinke, 2015; Bakar, 2016), there is a lack of research on moderators being cognitive in nature. This is significant because cognition is closely related to the innovative process (Wu et al., 2014; Martins et al., 2015). Thus, we have chosen cognitive adaptability as a moderator between the quality of mentoring top leaders and their perception of organizational innovativeness for several reasons. First, to be high in cognitive adaptability means one is prone “to be self-aware, to think aloud, to reflect, to be strategic, to plan, to have a plan in mind, to know what to know, to self-monitor” (Guterman, 2002). That is not only essential for innovating (Johnston and Bate, 2013), but is also part of what mentoring does to promote or develop such metacognition and self-regulation (Godshalk and Sosik, 2000). Second, this is a new area of study because no prior link between mentoring and any outcome has ever been examined under the moderating conditions of cognitive adaptability. Furthermore, we posit that mentoring is most advantageous for those mentees with lower levels of cognitive adaptability because if the mentee already has high cognitive adaptability, then the mentee may gain less from the mentoring experience because of redundancies in both what the mentor is trying to do and what the mentee naturally does (Haynie et al., 2012). Therefore, when top leaders with lower levels of cognitive adaptability are mentored, perceived organizational innovativeness increases at a greater rate from mentoring than those with a higher level of cognitive ability. Thus, this study explores the negative moderating effect of cognitive adaptability on the relationship between the quality of mentoring top leadership and perceived organizational innovativeness.

In summary, this study examines the value of mentoring top leadership and the conditions under which its relationship with perceived organizational innovativeness can be mediated by psychological safety and moderated by cognitive adaptability.

### Mentoring

Mentoring is a socially based learning process between mentor and mentee; since there are over 50 definitions in circulation (Crisp and Cruz, 2009), it is difficult to give a more precise definition (Dawson, 2014). For the purposes of this study, however, the focus will draw from Bandura and Walters’ (1977) social learning theory and emphasize the social dimension by defining mentoring as a social process for relevant knowledge transfer including formal or informal communication during a period of time “between a person who is perceived to have greater relevant knowledge, wisdom, or experience (the mentor) and a person who is perceived to have less (the protégé)” (Bozeman and Feeney, 2007). Furthermore, mentoring has three general functions from which the quality of mentoring can be measured: vocational support (coaching), psychosocial support (encouraging), and role modeling (demonstrating), each of which play an important role in developing an environment for innovative thinking and risk-taking (Scandura and Ragins, 1993; Godshalk and Sosik, 2000).

Mentoring is significant in the development of innovativeness for several reasons. First, Bandura (1986) states that discovering or learning new things in part relates to observing others within the context of social interactions and experiences (e.g., mentoring). Second, Bandura (2004) also teaches that learning by observation or social modeling enables the behaviors of others to serve as social prompts that activate, channel, and support modeled styles of behaviors or attributes (e.g., innovativeness). Likewise, since “innovation is a human-driven, social activity” (Anthony, 2012) and since mentoring in a business setting is not only a social interaction but also an opportunity to progress, we postulate mentoring top leaders has a positive relationship with their perception of organizational innovativeness. Therefore, we have developed the following prediction:

*Hypothesis 1:* The quality of mentoring top leaders will relate to their perception of organizational innovativeness.

### Psychological Safety

This study defines psychological safety as an individual’s belief to be safe without fear of negative results (Kahn, 1990). As explained previously, psychological safety can account for some of the influence mentoring has on innovativeness. Therefore, we have chosen perceived psychological safety of the organization as a mediator in the relationship of mentoring top leaders and their perception of organizational innovativeness for four reasons. First, psychological safety positively relates to mentoring because the mentoring functions of role modeling, psychosocial support, and vocational support (Scandura and Ragins, 1993) are essential resources for the development of psychological safety within the organization (Chen et al., 2014). For example, role modeling (demonstrating) heightens mentees’ psychological safety because the visual example can help motivate mentees to seek learning in the organization despite slip-ups (McCauley and Young, 1993). Furthermore, mentoring’s psychosocial support (encouraging or counseling) fosters psychological safety because it builds trust and a feeling that the mentee is cared about, allowing the mentee to emanate that feeling throughout the organization (Wang et al., 2010). Additionally, mentoring’s vocational support (coaching) encourages psychological safety by improving the mentee’s skills thereby reducing the probability of mistakes and engendering the mentee’s confidence that he or she can succeed in the organization (Kram and Isabella, 1985). Second, mentors purposefully assign difficult tasks to their mentees, train them, and provide feedback accordingly instead of chastisement as a way of challenging and growing their potential (Kram, 1983). Research shows that such supportive mentoring can create a climate where mentees feel psychologically safe in the organization (Edmondson, 1999; May et al., 2004; Nembhard and Edmondson, 2006).

Third, psychological safety relates to innovativeness because in order to innovate, one must try new things. Trying new things implies some form of risk taking. Consequently, the safer individuals feel the more likely they are to explore and discover (Edmondson, 2008). Fourth, as the level of psychological safety is developed within individuals in the organization, they will participate more in discussions and feel free to contribute their ideas because they spend less time regulating interpersonal relations (Edmondson, 1999). Many field studies also provide sound evidence on the positive relationship between psychological safety and discovering such things as new ideas, processes, and products in an organization (Cannon and Edmondson, 2001; Edmondson, 2003, 2004; Carmeli, 2007; Wong et al., 2010; Kostopoulos and Bozionelos, 2011; Hirak et al., 2012). Thus, we suggest that psychological safety is an appropriate mediator because it can become part of the bridge that links mentoring to innovativeness. For example, effectively mentoring top leaders can guide their perception of psychological safety within the organization, which then can influence their perception of organizational innovativeness. Therefore, we have developed the following prediction:

*Hypothesis 2:* The mentees’ perception of psychological safety in the organization mediates the relationship between the quality of mentoring they receive and their perception of organizational innovativeness.

### Cognitive Adaptability

In order to keep up with today’s unprecedented rate of innovation, some scholars have suggested that “successful future strategists will exploit an entrepreneurial mindset [which is] the ability to rapidly sense, act, and mobilize, even under uncertain conditions” (Ireland et al., 2003). Implied in the “entrepreneurial mindset” is that it is part cognitive in nature (Haynie et al., 2009).

Originally, in the field of entrepreneurship, cognition research was used to better understand opportunity recognition (McMullen and Shepherd, 2006). In a prior study, Haynie et al. (2009) embraced this endeavor but proposed to use the study of cognition in a new way to better explain the entrepreneurial mindset. In their study, they questioned why some entrepreneurs think differently about a given entrepreneurial task and credit the way they think as a key element of their success. Their study argued that the differences in performance of entrepreneurial tasks may in part be explained by the role that metacognition plays in promoting cognitive adaptability (Haynie et al., 2009). Hence, a foundational pillar of the entrepreneurial mindset is cognitive adaptability—the ability to be dynamic, flexible, and self-regulating in one’s cognitions in given dynamic and uncertain task environments.

Successful executives or entrepreneurs (and therefore their companies) are swift to modify and adjust depending on what they discover, rather than stubbornly persisting regardless of the feedback or data they receive. Obviously, entrepreneurs and executives need to be persistent but the principle of persistence can often be misapplied when pursing a less innovative path for the sake of not quitting. Top leaders need to develop the ability to recognize new information and then change the way they view the world in order to create something different that has value. Cognitive adaptability can help entrepreneurs and executives alike to do just that: become more innovative, and thus lead their organizations to do and become likewise (Furr and Ahlstrom, 2011).

We have chosen to study cognitive adaptability as a moderator between the quality of mentoring top leaders and their perception of organizational innovativeness for at least three reasons. First, cognitive adaptability is linked to mentoring because mentoring fosters the kind of self-reflection and metacognition that results in cognitive adaptability (Godshalk and Sosik, 2000). Second, it is linked to innovativeness because cognitive adaptability creates the kind of strategic thinking that is crucial for innovating (Johnston and Bate, 2013). Third, since cognitive adaptability may be more natural in some but may need to be developed (through mentoring) in others (Haynie et al., 2012), we put forward that the quality of mentoring top leaders has a different effect on their perception of organizational innovativeness depending on the level of cognitive adaptability in the leader (mentee).

We predict that effectively mentoring top leaders has its greatest impact on their perceived organizational innovativeness when cognitive adaptability is low. Top leaders already high in cognitive adaptability (i.e., they are highly dynamic, flexible, and self-regulating) gain less through quality mentoring because of redundancies in both what the mentoring functions do and what the mentee naturally does; thus, affecting their perception of innovativeness at a less dramatic rate. Likewise, when top leaders with lower levels of cognitive ability are effectively mentored, perceived organizational innovativeness increases at a greater rate than in those with a higher level of cognitive ability. Indeed, it is likely that top leaders low in cognitive adaptability would be less willing to take advice from a mentor because they are not as flexible, thereby not changing behavior. However, all leaders in this study have received mentoring, which suggests that they at least had a desire to move forward in some way and may not be as rigid as that argument implies. Those with low cognitive adaptability are possibly just less experienced or less natural in that ability. Thus, we suggest that if high levels of the mentoring functions mentioned previously occur (vocational and psychosocial support and role modeling—Scandura and Ragins, 1993), then the leader with low levels of cognitive adaptability is affected at steeper rate due to the contrast with their typical lower self-regulating state. Therefore, we have developed the following prediction:

*Hypothesis 3:* The top leaders’ perception of organizational innovativeness increases at a greater rate when the quality of mentoring is high and the cognitive adaptability is low.

**FIGURE 1:**
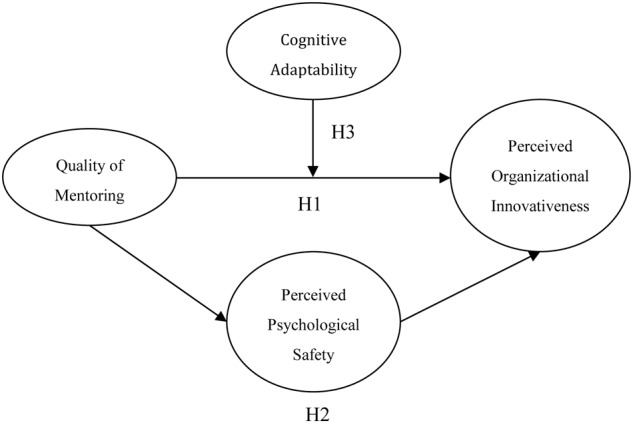
**Conceptual Model and Hypotheses**.

**Figure 1** illustrates the conceptual model for this research.

## Materials and Methods

### Sample and Procedure

We used organizations’ and individuals’ executive networks to search, screen, and select candidates who met several criteria also used by Baron (2008). First, participants needed to be those who personally identify and exploit opportunities rather than those who do so merely as part of a team. Since we focused on the individual level of analysis and in order for the findings to have any relevance and reliability, the candidates needed to be those who have high potential influence on the organization’s level of innovativeness and be in an appropriate position to accurately judge or perceive organizational innovativeness. Second, they needed to have been mentored. Third, participants needed to have a higher level title, such as founder, president, CEO, entrepreneur, etc.

The screening and data collection took place over 6 months, after which approximately 303 candidates qualified for the study. Only 200 participated by taking our questionnaire (a 66 percent response rate). Because of the global reach of these executive networks, the participants came from 14 countries; 43 percent from Western countries and 57 percent from Asia. The countries include Canada, China, Cuba, France, Hong Kong, Japan, Korea, New Zealand, The Netherlands, Singapore, Switzerland, Taiwan, The United Kingdom, and The United States. Women represent 18.5 percent of the sample. The mean age of participants was 37.98 (*SD* = 10.84). The average length of tenure in the organization’s top leadership position was 5.35 years (*SD* = 6.44). The participants’ organizations had been established for an average of 20.2 years (*SD* = 28.15). Approximately 39 percent of the organizations had over 100 employees and the organizations represented approximately 45 different industries such as healthcare, textiles, real estate, and education. The mentors of these executives were typically members of the board of directors or were outside experts.

### Measures

All measures used Likert-type scales. Both English and Chinese-language versions of the questionnaire were used to collect data. In the People’s Republic of China, the questionnaire was translated from English to Chinese using a conventional back-translation procedure (Brislin, 1980).

#### The Quality of Mentoring

The items were adapted from prior research so they could be worded in context with the goals of our study. Three items were used to measure mentoring’s vocational support function from Scandura and Ragins (1993; “Mentor has devoted special time and consideration to my company,” “My mentor provided me with challenges to improve,” and “Mentor gives me special coaching on the job of an executive”). The psychosocial support function of mentoring was measured using two items from Tharenou (2001; “I socialize with my mentor after work” and “I exchange confidences with my mentor”). The role modeling function of mentoring was measured using two items from Noe (1988; “I respect and admire my mentor” and “I agree with mentor’s attitudes and values”). All items were measured on a 1-to-5 strongly disagree-strongly agree scale. The fit indices for three first-order factors plus 1 s-order factor fell within an acceptable range (χ^2^ [11, *n* = 200] = 18.69, *p* < 0.001; TLI = 0.97, CFI = 0.98, RMSEA = 0.06), indicating that these dimensions were distinctive and the overall construct was collectively reflective. The Cronbach’s alpha was 0.82.

#### Organizational Innovativeness

It was measured using a five-point scale from Hurley and Hult (1998) consisting of five items. Sample items were “People are not penalized for new ideas that do not work,” “Innovation in our company is encouraged,” and “We actively seek innovative product and service ideas.” The Cronbach’s alpha was 0.91.

#### The Psychological Safety of the Organization

It was measured using an eight-item scale developed by Anderson and West (1994). The scale was originally designed to assess team climate and was used in this study over other more common psychological safety measures because the wording was more broad and applicable to top leaders assessing the psychological safety of the organization. Sample items were “People feel understood and accepted by each other,” “Everyone’s view is listened to, even if it is in a minority,” “We have a ‘we are together’ attitude,” and “There is a lot of give and take.” The Cronbach’s alpha was 0.91.

#### Cognitive Adaptability

It was measured using a shortened version of the cognitive adaptability scale from Haynie and Shepherd (2009), because the full scale would have made the instrument onerously long for time-constrained CEOs or equivalents and possibly introduce its own bias into the results. The original scale has 35 items measuring five dimensions of cognitive adaptability; the 10-item scale used in this study was composed of two items with highest loadings from each of the five dimensions. Sample items were “I often define goals for myself,” “I know what kind of information is most important to consider when faced with a problem,” “I think of several ways to solve a problem and choose the best one,” “I ask myself if I have considered all the options after I solve a problem,” and “I stop and re-read when I get confused.” A Likert-type scale was used on all the items (1 = “not very much like me” to 10 = “very much like me”). The Cronbach’s alpha was 0.89.

Given that we did not use the full version of the scale, we assessed the validity of the shortened scale by comparing these two versions of the scale using an independent field sample where participants evaluated their cognitive adaptability by using the full version of the scale. We obtained a sample of 183 MBA students (57 percent response rate) who rated themselves using the 35-item cognitive adaptability scale. We also asked them to evaluate their individual innovative behavior using Yuan and Woodman’s (2010) 6-item scale. The correlation between the 10-item and 35-item scale was 0.85 and the *α*’s for the 10- and 35-item scales were 0.71 and 0.86, respectively. We also tested the relationship between cognitive adaptability and individual innovative behavior to assess the comparative criterion-related validity, comparing the 10- and the 35-item scales. The 10-item scale was significantly related to individual innovative behavior [*R^2^* = 0.12, *F*(1,181) = 25.23, *p* < 0.01; *β* = 0.24, *p* < 0.01] as well as the full 35-item scale [*R^2^* = 0.19, *F*(1,181) = 44.27, *p* < 0.01; *β* = 0.38, *p* < 0.01].

### Analytic Strategies

We ran a confirmatory factor analysis at first to test the model fit by AMOS 22.0, adopting five widely reported and recommended indices (Hu and Bentler, 1999; Kline, 2011). The mediating effect and moderating effect were tested by bootstrap methods, using PROCESS macro (version 2.15), which was originally developed by Hayes (2013).

### Ethics Approval Statement

Even though our study was not in the area of medical research and the data in our study was voluntarily self-reported, we followed applicable research procedures in accordance with the Helsinki Declaration as revised in 2013. Our research was approved by Zhejiang University’s Global Entrepreneurship Research Center Committee. All participants in our study were provided sufficient information to be able to give an informed consent to take part in this study. Research respondents were ensured confidentiality and anonymity. All participation was voluntary. We confirm this research is independent and impartial.

## Results

**Table 1** presents summary statistics and bivariate correlations among the variables.

**Table 1:** Descriptive statistics and intercorrelations of variables.

	Pearson correlations								
	Mean	*SD*	1	2	3	4	5	6	7
(1) Age	37.98	10.84							
(2) Gender	0.19	0.39	0.04						
(3) Tenure	5.35	6.44	0.63^∗∗^	0.02					
(4) Quality of mentoring	3.43	0.66	0.17^∗^	-0.19^∗∗^	0.17^∗^	**0.82**			
(5) Perceived psychological safety	3.77	0.71	0.18^∗^	-0.07	0.13	0.47^∗∗^	**0.91**		
(6) Cognitive adaptability	7.35	1.39	0.31^∗∗^	0.01	0.25^∗∗^	0.45^∗∗^	0.64^∗∗^	**0.89**	
(7) Perceived organizational innovativeness	3.78	0.82	0.34^∗∗^	-0.15^∗^	0.18^∗^	0.45^∗∗^	0.45^∗∗^	0.45^∗∗^	**0.91**

*n = 200. Internal consistency coefficients are reported in bold on the diagonal. Gender was recorded as male = 0 and female = 1. The control variables were not significant. ^∗^*p* < 0.05, ^∗∗^*p* < 0.01.*

### Confirmatory Factor Analysis

We ran a confirmatory factor analysis to test whether our hypothesized model captured distinct constructs. The results show that the hypothesized 4-factor model fits the data acceptably and the hypothesized model captures distinct constructs, with χ^2^ [203, *n* = 200] = 416.54, CFI = 0.92, TLI = 0.91, RMSEA = 0.07, and SRMR = 0.06.

### The Mediating Role of Psychological Safety

**Table 2** presents the results of a regression analysis of the mediating effect (all coefficients are unstandardized). As shown in **Table 2**, the total effect of the quality of mentoring on organizational innovativeness is significantly positive (*b* = 0.56, *p* < 0.001), thus supporting Hypothesis 1. **Table 2** also presents the direct effect of the quality of mentoring on organizational innovativeness. We also found that the model fit of this mediating effect is acceptable [*R^2^* = 0.20, *F* (1,198) = 51.50, *p* < 0.001].

**Table 2:** The regression analysis of mediating effect.

Effect	Variable	Effect	SE
Direct effect of X on M	Perceived psychological safety	0.51^∗∗∗^	0.07
Direct effect of M on Y	Perceived organizational innovativeness	0.34^∗∗∗^	0.08
Total effect of X on Y	Perceived organizational innovativeness	0.56^∗∗∗^	0.08
Direct effect of X on Y	Perceived organizational innovativeness	0.39^∗∗∗^	0.09

*n = 200. X, independent variable (the quality of mentoring), Y, dependent variable (perceived organizational innovativeness), M, mediator (perceived psychological safety). ^∗∗∗^*p* < 0.001.*

We adopted bootstrap methods to test the mediating effect by SPSS PROCESS macro (version 2.15), which is concerned with indirect effect (Shrout and Bolger, 2002). We also test the mediating effect by expecting the indirect effect would be non-zero (MacKinnon et al., 1995). We find that the indirect effect of the quality of mentoring on organizational innovativeness through psychological safety is 0.17 (95% CI [0.0846, 0.2781]). With the confidence interval excluding zero, thus Hypothesis 2 is supported.

### The Moderating Role of Cognitive Adaptability

**FIGURE 2:**
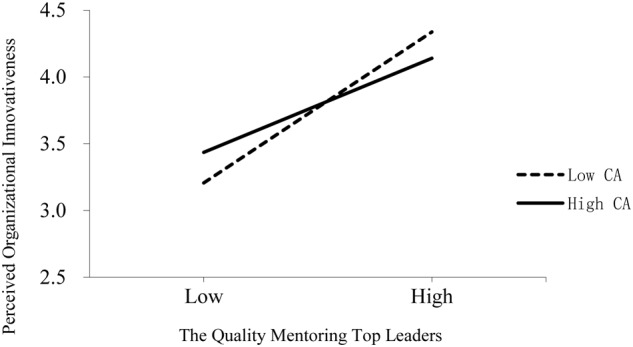
**Interactive effects of the quality of mentoring top leadership and their cognitive adaptability on perceived organizational innovativeness**.

The moderating effect was also tested by SPSS PROCESS macro (version 2.15). The coefficient of XW on Y was –0.13 (95%CI [-0.2468, -0.0132]), showing that cognitive adaptability negatively moderates the effect of mentoring on innovativeness. Therefore, Hypothesis 3 is supported. The conditional effect of mentoring on innovativeness was computed by PROCESS, as shown in **Table 3**. The conditional effect varies at different levels of cognitive adaptability (-1 SD as Low: 5.97; +1 SD as High: 8.74). **Figure 2** displays the interactive effects of the quality of mentoring and cognitive adaptability on perceived organization innovativeness, which shows that when top leaders with lower levels of cognitive ability are effectively mentored, perceived organizational innovativeness increases at a greater rate from mentoring than those with a higher level of cognitive ability.

**Table 3:** The moderating effect of the quality of mentoring on perceived organizational innovativeness.

Outcome	Moderator	Effect	SE	95% CI
Perceived organizational	Low	0.61	0.13	[0.3537, 0.8737]
innovativeness	High	0.25	0.10	[0.0506, 0.4560]

## Discussion

This study confirms that the quality of mentoring top leaders does positively relate to their perception of organizational innovativeness and that the relationship is mediated by their perception of psychological safety within the organization and negatively moderated by their cognitive adaptability. These outcomes offer theoretical and managerial implications. Limitations are also discussed.

### Theoretical Implications

Recent empirical studies (Orpen, 2010, 2013; Rollins et al., 2014; Mitchell et al., 2015) consider mentoring at lower level employee outcomes but neglect higher level leader outcomes, which confirms previous statements that “very little is known about the nature of [mentoring executive] relationships” (Clutterbuck and Megginson, 1999). The purpose of this study has been to shed more light on such an influential subject and has done so in several ways. First, in addition to former studies finding that mentoring is related to several positive outcomes (Wanberg et al., 2006; Mitchell et al., 2015), this study adds the additional outcome of perceived organizational innovativeness, which is significant given the growing need for innovative leaders and organizations today (Pisano, 2015). Second, given that higher level leaders are different from those at lower levels in terms of their high position, responsibility, and potential to influence the organization (Papadakis and Barwise, 2002; Conger and Fulmer, 2003), this study leads to a better understanding of mentoring’s influence on top leadership. Third, although this study does not investigate the differences between mentoring higher level leaders and lower level employees, it does show that the quality of mentoring matters at higher level positions. Lastly, this study answers the call for more research on mentoring higher level leadership (de Janasz and Peiperl, 2015) and confirms the continuing need to learn more about developing this influential demographic.

In addition, this study responds to the call for more research on the mediating influence of psychological safety in mentoring (Chen et al., 2014) and gives convincing evidence that effective mentoring of top leaders can help promote their perception of psychological safety within organizations thereby heightening their perception of organizational innovativeness, which is a new finding in current research. Furthermore, this research responds to other calls for a better understanding of how cognitive adaptability interacts with other factors (Haynie and Shepherd, 2009; Haynie et al., 2009, 2012). We have shown that when top leaders with lower levels of cognitive adaptability are effectively mentored, perceived organizational innovativeness increases at a sharper rate than those with a higher level of cognitive adaptability, which is also a unique finding in today’s literature. Finally, this study addresses recent calls for examining mentoring relationships in a more global context and not just in western cultural contexts by including participants from 14 countries, 57 percent coming from Asia. Therefore, this study is able to generalize findings for other cultures and promote cross-cultural research (Bozionelos and Wang, 2007).

### Managerial Implications

The findings from this research have practical implications as well. Innovation is “the source of sustained advantage for most companies” and depends upon the individual expertise of those who lead the organization (Leonard and Sensiper, 1998). In that light, and in seeing the positive relationship between mentoring and organizational innovativeness, it is clear that it is critical for organizations to have a mentoring program in place (Allen et al., 2009; Liu et al., 2012), especially among those who have the most influence within the organization. Therefore, it is imperative for organizations to design effective formal (organization-appointed mentors) or informal (mentee-appointed mentors in or outside the organization) mentoring programs for top leadership in order to ensure high-quality mentoring relationships. Within these purposeful formal or informal mentoring programs for higher level leadership, it is also essential for the selected mentors to understand the goals and purposes of the mentees or programs in order to be most effective (Allen et al., 2006). Thus, organizations could offer preparation programs for mentors that would increase their interest as well as their ability to help the organization become more innovative.

This study also helps spotlight the vital role psychological safety plays within the organization. According to social learning theory, people are social creatures and learn by observation and repeat what they learn (Bandura and Walters, 1963). As top leadership is effectively mentored through vocational support, psychosocial support, and role modeling, confidence is enhanced, resulting in less fear of making mistakes and the willingness to embrace risk. This newfound confidence can begin to influence the goals, plans, and eventually the whole organization. One of, if not the biggest advantages of feeling more psychologically safe as a leader is the realization that the organization needs to make allowances for some mistakes in order for members of the organization to perceive psychological safety themselves and be willing to try out new ideas. In summary, top leaders need to feel psychologically safe so that they will be more innovative and leaders need to understand that a feeling of psychological safety will lead to increased innovation throughout the organization.

The results regarding the negative moderating role of cognitive adaptability suggest that organizations need to be able to identify those leaders who have the most need of mentoring, in other words those who are less cognitively adaptable, either through inexperience or because of natural tendencies. Valuable resources are usually limited and so it requires a careful assessment of where appropriate executive leadership mentors should be assigned. Additionally, those executives or entrepreneurs who choose their own mentor, should choose one who is an expert and different enough from themselves in order to assist top leaders to become more dynamic, self-aware, strategic, and flexible.

Finally, in a business environment where the only thing constant is change, individuals and organizations need to learn how to embrace change and transform their culture in order to innovate at today’s unprecedented rate. Given individuals naturally tend to resist change in organizations because it can be a painful process (Marquis and Tilcsik, 2013), this study suggests that mentoring top leaders is a potential way to help facilitate transforming leaders and thereby organizational culture to becoming more innovative.

### Limitations

Notwithstanding this study’s implications and contributions, it has a number of limitations. First, this study was limited in testing the causal inferences between variables by using a cross-sectional design. A longitudinal design would be more ideal for examining how mentoring top leadership works within different time periods. Second, the data is self-reported, which brings with it several common criticisms. However, although the data is self-reported, using top leadership ratings as the measures has advantages because such ratings are usually based on a broader perspective that comes with the higher level position and arguably commands the most weight in terms of judgment. Third, it can be debated that in many circumstances, whether or not an organization is innovative depends upon situational dynamics outside the CEO or entrepreneur’s control, which an individual mentor is also not likely to be able to change or alter. However, although organization innovativeness can be influenced by factors beyond the leadership’s control, it can also be said that CEOs or entrepreneurs from the top down have the ability to impact organizational innovativeness.

## Conclusion and Future Research

In conclusion, this study reveals three main findings. First, the quality of mentoring top leaders is linked to their perception of organizational innovativeness, which means effective mentoring is one potential way to increase innovativeness. Second, top leaders’ perception of psychological safety within the organization mediates the relationship between the quality of mentoring and their perception of organizational innovativeness, which means effective mentoring is another way to improve innovativeness via the development of psychological safety. Third, cognitive adaptability negatively moderates the relationship between mentoring and innovativeness, which means effective mentoring can potentially help leaders with lower levels of cognitive adaptability foster innovativeness within the organization. As a result, this study demonstrates the value of mentoring top leadership. Furthermore, this study advocates that it is important to establish a psychologically safe environment to inspire not only top leadership to try new avenues but also to encourage/enable all those within the organization to feel free to express their opinions and ideas. Additionally, our findings encourage organizations to proactively and selectively prioritize mentoring among top leadership taking into consideration their differing levels of cognitive adaptability. Future studies should examine the differences between higher and lower level mentoring and the strategies that cause mentoring top leadership to be effective. Finally, further research could focus on how to provide greater support to the mentors of top leaders.

## Author Contributions

All authors listed, have made substantial, direct and intellectual contribution to the work, and approved it for publication.

## Conflict of Interest Statement

The authors declare that the research was conducted in the absence of any commercial or financial relationships that could be construed as a potential conflict of interest.
